# A Review of Sensing Technologies for Indoor Autonomous Mobile Robots

**DOI:** 10.3390/s24041222

**Published:** 2024-02-14

**Authors:** Yu Liu, Shuting Wang, Yuanlong Xie, Tifan Xiong, Mingyuan Wu

**Affiliations:** School of Mechanical Science and Engineering, Huazhong University of Science and Technology, Wuhan 430074, China; yu_liu_d@hust.edu.cn (Y.L.); wangst@hust.edu.cn (S.W.); Xiongtf@hust.edu.cn (T.X.); mingyuan_wu@hust.edu.cn (M.W.)

**Keywords:** sensing technology, SLAM, obstacle avoidance, sensor fusion

## Abstract

As a fundamental issue in robotics academia and industry, indoor autonomous mobile robots (AMRs) have been extensively studied. For AMRs, it is crucial to obtain information about their working environment and themselves, which can be realized through sensors and the extraction of corresponding information from the measurements of these sensors. The application of sensing technologies can enable mobile robots to perform localization, mapping, target or obstacle recognition, and motion tasks, etc. This paper reviews sensing technologies for autonomous mobile robots in indoor scenes. The benefits and potential problems of using a single sensor in application are analyzed and compared, and the basic principles and popular algorithms used in processing these sensor data are introduced. In addition, some mainstream technologies of multi-sensor fusion are introduced. Finally, this paper discusses the future development trends in the sensing technology for autonomous mobile robots in indoor scenes, as well as the challenges in the practical application environments.

## 1. Introduction

In recent times, there has been a rapid development in autonomous mobile robots (AMRs). Due to their excellent work ability, AMRs are being used to replace humans in some indoor scenarios. Mobile robots can help reduce the burden of human work and improve production efficiency. They have been widely used in many industries and services, such as warehousing, logistics, healthcare, restaurant service, and personal services [1,2,3].

The basics of mobile robotics consist of the fields of locomotion, perception, cognition, and navigation [4]. The locomotion problem is mainly concerned with the motion system of the mobile robot. The motion system design is based on the requirements of the provided services, such as the motion environment, controllability, efficiency, stability, and other relevant indicators. Perception involves acquiring information about the mobile robot’s working environment and itself. Cognition involves analyzing and processing data from the perception system and providing control solutions to the motion system to accomplish the mobile robot’s tasks. Mobile robot navigation relies on perception, cognition, and motion control to move from a starting point to a task goal point in a work environment, whether known or unknown. Perception is a crucial part of mobile robotics research. For mobile robots to safely and efficiently perform mobile tasks, they must sense their environment and make decisions based on that information. If a mobile robot cannot perceive its environment accurately and efficiently, then it will not be able to perform even simple tasks [5].

The perception system of the mobile robot utilizes relevant sensing techniques to provide information about the environment, the robot itself, and the relationship between the two. This information is then used for path planning, controlling the robot’s movement, and ultimately completing the navigation task. The main tasks of the perceptual system include localization, map building, object detection, etc.

**Figure 1:**
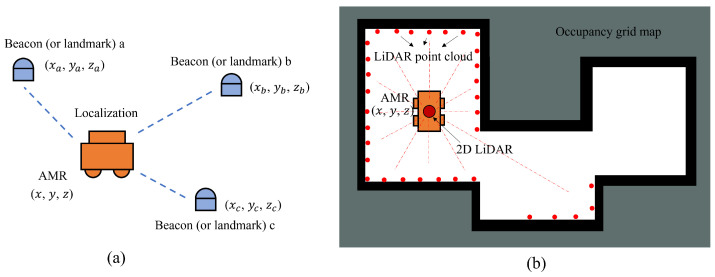
Schematic diagram of the localization method: (**a**) the localization method base on active beacon or landmark, and (**b**) the localization method base on model matching of 2D LiDAR.

Localization is the process of estimating a mobile robot’s position relative to the surrounding work area during its movement. This information serves as the foundation for the mobile robot’s navigation [6,7,8]. Mobile robot localization techniques include inertial navigation, global positioning systems (GPS), active beacon-based navigation, landmark navigation, and model matching. Inertial navigation is a navigation method that utilizes data from sensors responding to inertial forces. The global positioning system (GPS) is a satellite-based technology that provides accurate information on longitude, latitude, and altitude for any location on Earth. It is a highly effective technique for locating mobile robots in outdoor environments. However, GPS is of limited use for localizing in indoor environments, due to non-line-of-sight and signal-blocking problems in complex environments [9]. Active beacon-based navigation and landmark navigation use relative positional relationships to beacons or landmarks with known locations in the environment for localization, as shown in Figure 1a. Model matching is a method of positioning by matching sensor data with specific map model data. For instance, in 2D LiDAR localization, the LiDAR measurement obtains 2D laser point cloud data, and the map model is an occupancy grid map. The mobile robot localization is performed by matching the point cloud data with the occupancy grid map, as shown in Figure 1b.

**Figure 2:**
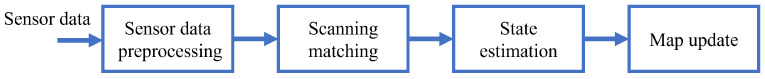
Schematic diagram of the SLAM method based on filter.

Map building typically involves Simultaneous Localization and Mapping (SLAM), which is a crucial research field for mobile robots. SLAM is a technique used by mobile robots to determine their location in an unknown environment while simultaneously constructing an accurate map of the environment. The localization component estimates the robot’s position on an existing map, while the mapping component constructs the environment’s map [10,11,12]. The two components are interdependent in completing the map building process and enhancing accuracy through continuous iteration. The two most frequently used sensors in SLAM are LIDAR and vision cameras. SLAM techniques can be classified into two main categories based on short- and long-time optimization processing: filter-based methods and graph optimization-based methods. Filter-based SLAM algorithms typically use various types of filters to estimate and optimize the robot’s trajectory and map, as shown in Figure 2. The two most common filter-based algorithms are the Kalman filter SLAM algorithm and the particle filter SLAM algorithm.

**Figure 3:**
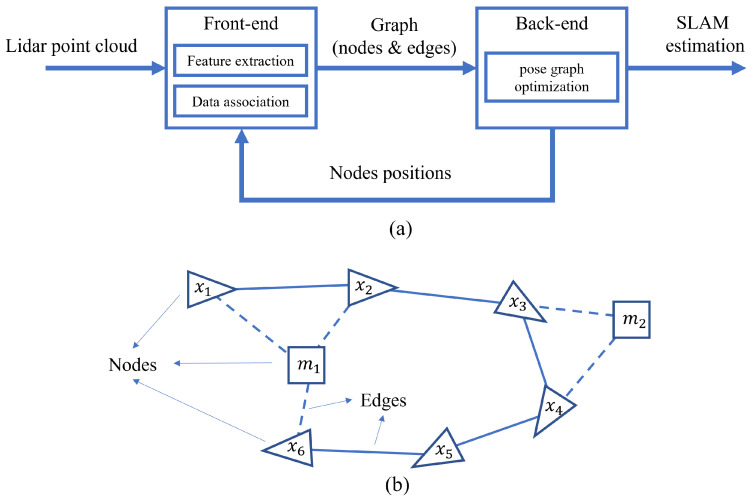
Schematic diagram of the SLAM method based on graph optimization: (**a**) representation of a graph optimization SLAM process, and (**b**) schematic representation of the graph optimization algorithm.

The graph optimization algorithm mainly consists of two processes, front-end and back-end, as shown in Figure 3a. The graph optimization algorithm models the robot’s trajectory and the environment’s topology as a graph, and minimizes the error by optimizing the nodes and edges in the graph to estimate the robot’s position and obtain a mao of the environment, as shown in Figure 3b. Nodes generally represent the variables to be optimized in the SLAM process of the sensor, such as sensor position, feature point location, estimated trajectory, etc. Edges represent the errors or constraints between the variables. The optimization algorithm is iterated to minimize the error of the edges to find the optimal solution [13].

With the development of artificial intelligence, the emergence of reinforcement learning gradually allows mobile robots to gradually discard the limitations associated with a priori maps, and achieve the judgement of navigation actions only through image ingestion [14,15]. The main logic is: to set up a series of exploration reward mechanisms around the mobile robot exploration problem, such as: arrival reward, collision reward, curiosity reward, etc., and to establish the relevant neural network through reinforcement learning methods, and to train the network using the data set. Finally, the navigation decision of the autonomous mobile robot is realised.

To accomplish the navigation task efficiently and accurately, the mobile robot must avoid colliding with obstacles. Obstacle avoidance is a crucial task for autonomous mobile robots moving in uncertain environments [16,17]. Obstacle avoidance focuses on the problem by obtaining the global position information of the robot relative to the surrounding environment to the mobile robot only needs to obtain the local position information of the close objects around the robot relative to itself. Obstacle detection is crucial for perception systems as well. The mobile robot’s cognition system performs path planning to avoid obstacles based on the distance and direction information about obstacles obtained from the perception system. In principle, range sensors (ultrasonic sensors and LiDAR) are highly suitable for obstacle detection tasks. In addition, vision cameras can recognize obstacles in the environment and even predict the trajectory of dynamic obstacles.

The perception system achieves these functions through the use of sensors and extracting relevant information from their measurements. Sensors used for data collection are categorized into two major aspects: proprioceptive/exteroceptive sensors and active/passive sensors [3]. Proprioceptive sensors gather data about the interior of the mobile robot, such as motor speed, wheel rotation angle, etc. Currently, encoders [18], accelerometers, and gyroscopes [19] are widely used in this type of sensor. Exteroceptive sensors acquire information about the environment around the mobile robot, such as images, sound waves, and wireless signals. Examples of exteroceptive sensors include vision sensors [20], ultrasonic sensors [21], and LiDAR [22]. Active sensors send energy to the external environment and then measure the environmental feedback, such as sonar sensors, LIDAR, etc. Passive sensors measure the environmental energy coming into the sensor, such as vision sensors (Charge Coupled Device (CCD) or Complementary Metal Oxide Semiconductor (CMOS) cameras).

This paper describes the functions and principles related to the perception system of mobile robots. Further, this paper analyzes the application of various types of sensors in sensing technology. Additionally, the research results for multi-sensor fusion are presented here. The rest of this article is organized as follows: Section 2 describes some extensively adopted sensors; Section 3 presents the mainstream multi-sensor fusion schemes; and Section 4 discusses perception techniques and potential technology trends.

## 2. Overview of Single Sensor Sensing Technologies

### 2.1. Inertial Measurement Unit (IMU)

**Figure 4:**
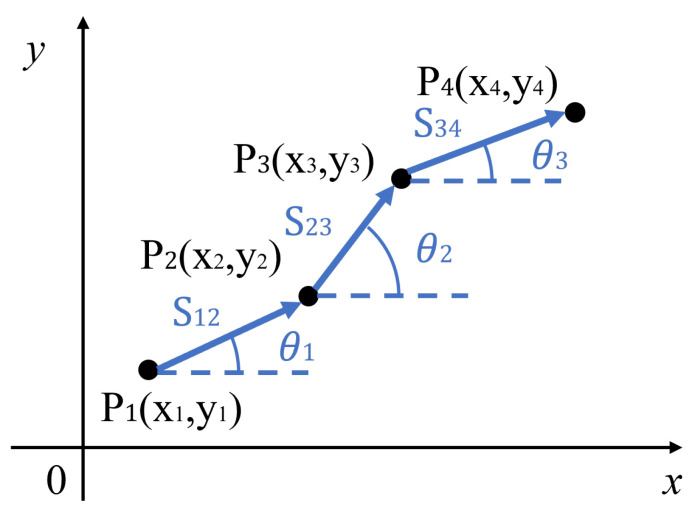
IMU dead reckoning.

An IMU is an electronic device that integrates an accelerometer, gyroscope, and magnetometer to measure an object’s acceleration, angular velocity, and the direction of the geomagnetic field [23]. The IMU is the proprioceptive sensor. The Inertial Navigation System (INS) is a dead-reckoning navigation system that uses the Inertial Measurement Unit (IMU) and is commonly used for mobile robot navigation. Magnetometer-measured orientation parameters have low accuracy and are challenging to use for precise navigation tasks. Therefore, the most frequently used parameters in IMUs are acceleration and angular velocity. The principle of dead reckoning is demonstrated in Figure 4. By using the considered IMU, the trajectory of an indoor AMR is determined by integrating the acceleration and angular velocity based on the initial position, direction, and velocity.

An IMU has two typical advantages: a high output update rate and immunity to external interference. These advantages are irreplaceable when a high data update rate or high reliability are required, or when the external signal acquired by the mobile robot system is unreliable.

However, IMUs have limitations when applied to mobile robots. IMUs have two typical disadvantages:

1. The calculation process must depend on the initial conditions.
2. The navigation error increases with time, which is due to the need for double integration of its measurements.

It should be mentioned that its duration and sensor accuracy have a significant impact on the accuracy of IMU-based navigation [23]. To overcome these disadvantages, an IMU is frequently used in fusion with other sensors and as auxiliary measurements in other navigation methods. L. Luo et al. [24] designed a positioning method that combines an IMU, Bluetooth, and a magnetometer for the mobile robot positioning problem. In the above-mentioned method, the inertial navigation system (INS) serves as the core, and the Bluetooth AOA positioning base station observes the position, while the magnetometer observes the heading angle. Alatise M. B. et al. [25] fused IMU and vision sensors in order to achieve accurate positioning of a mobile robot. The accelerometer data are used as inputs to the control, while gyroscope and image data are used as observations for position pose estimation using extended Kalman filtering. Zhang et al. [26] developed an algorithmic framework for the indoor localization of mobile robots by combining a wheel odometer, an inertial navigation unit (IMU), and Ultra-Wideband (UWB). The framework uses a fusion method with extended Kalman filtering, where the wheel odometer provides localization information as a prediction and the IMU and UWB provide attitude angle and position information as observations.

### 2.2. Ultrasonic Sensor/Sonar

**Figure 5:**
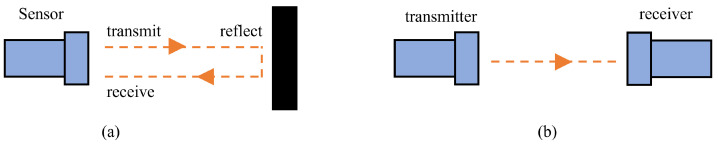
Sensor distance measuring method: (**a**) reflection-type distance measuring method. (**b**) unidirectional distance measuring method.

Ultrasound refers to sound waves that vibrate at frequencies above 20 kHz, which are beyond the upper limit of human hearing. Sonar also employs ultrasound. Ultrasonic sensors are typically distance measurement sensors. Distance measurement sensors can be classified into two types of measurement methods: the reflection-type distance measuring method and the unidirectional distance measuring method, as shown in Figure 5. In the reflection-type method, the sensor sends a signal to the surrounding environment, which is then reflected when it encounters an obstacle. The sensor then receives the reflected signal to measure the distance between the sensor and the obstacle. The unidirectional distance-measuring method involves placing the transmitter and receiver in different locations. The receiver measures the distance between the two by accepting the signal from the transmitter. Ultrasonic ranging measures distance using three main criteria: time, phase, and acoustic vibration.

Ultrasonic sensors typically use a reflection-type distance measuring method to determine the distance to an object, which is used for mobile robot positioning and navigation. However, the sonar sensor’s ultrasound beam width is too wide to precisely determine the object’s direction. Additionally, specular reflection on object surfaces often results in inaccurate distance calculations, reducing the reliability of distance measurements from sonar sensors. It is not possible to construct a complete grid map using erroneously measured sonar data when the frequency of erroneous distance measurements is too high. To overcome this problem, S.-J. Lee et al. [27] designed a method for constructing occupancy grid maps for mobile robots using sonar data. This method assigns weights to each sonar data point based on its geometric reliability to minimize the impact of incorrect sonar data on the grid map construction. Anomalous sonar data are identified and assigned a lower geometric reliability value through a morphological comparison with neighboring sonar data. H. Liu et al. [28] used sonar sensors to overcome the limitations of sensor performance by accumulating sonar data to achieve room-level localization. Y. Liu et al. [29] presented a mobile robot on the basis of LEGO MINDSTORM NXT, in which an ultrasonic sensor was mounted on a 360° rotating motor and a particle-filtering approach was used to implement its localization function. Mingqi Shen et al. [30] provided a localization method for autonomous mobile robots based on multiple ultrasonic sensors. The method calculates the target’s coordinates based on the ratio of three flight times measured by three ultrasonic sensors. This improves the localization accuracy by avoiding the effect of ambient temperature on localization.

In addition, ultrasonic sensors can also be used for the unidirectional distance measuring method, which has been applied in the localization of indoor mobile robots. R. Li et al. [21] set up an ultrasonic receiver on the robot and had multiple ultrasonic generators in the scene. The three-sided localization principle is used to achieve the localization of the mobile robot. Chen-Chien Hsu et al. [31] presented a localization method based on omnidirectional ultrasonic sensing. The proposed method utilizes reflective cones to generate a 360° propagating ultrasonic beam to address the detection angle limitation of conventional ultrasonic sensors. Ultrasonic waves are emitted directly onto a cone, which reflects them in all directions. The ultrasonic signal is received by the installed receiver in the environment. The mobile robot’s position is determined through the ToF (time-of-flight) method and the trilateration localization method.

Ultrasonic sensors are susceptible to environmental noise and long-range strength degradation [32] and are not accurate enough to measure detailed features of the environment. There are relatively few cases where ultrasonic sensors are used for positioning in high-precision indoor navigation applications. However, ultrasonic sensors have a noticeable advantage in obstacle detection. They are highly accurate in detecting highly transmissive or reflective objects, such as glass and mirrors, which can be challenging for many optical sensors. H. Takai et al. [33] used ultrasonic stereo sonar and a single image sensor to detect obstacles in the workspace. J.-H. Jean et al. [34] mainly used the environmental distance information collected by ultrasonic sensors to guide the robot along the wall baseline using a steering controller based on the potential field method. Grami T. et al. [35] designed a mobile robot equipped with seven ultrasonic sensors and used a particle-filtering approach to implement its localization function. The mobile robot can move freely while avoiding obstacles. Maryna Derkach et al. [36] utilized four ultrasonic sensors for obstacle avoidance in mobile robots. Their proposed algorithm uses a linear recursive Kalman filter to process the sensor data, allowing the robot to avoid additional obstacles.

### 2.3. Infrared Sensor

Infrared is an electromagnetic wave with a wavelength between visible light and microwaves. Infrared sensors use an emitter to emit an infrared beam and a receiver to measure the intensity of the beam. The intensity of the infrared beam decreases as the distance traveled increases. Infrared sensors can be used to measure distance by assessing the intensity of the beam, in addition to the ToF (time-of-flight) method. Similar to ultrasonic sensors, infrared sensors use reflection-type and unidirectional distance measuring methods.

There are many early studies applying infrared sensors to mobile robot localization. Eric Brassart et al. [37] designed a mobile robot localization system based on infrared beacons. In this system, a ceiling-mounted infrared beacon sends an encoded infrared signal, which is then received by a CCD camera mounted on the mobile robot. The robot’s location is determined using triangulation and trilateration methods. J. Krejsa et al. [38] presented an interior localization method for mobile robots that uses infrared beacons. The beacons are placed at known locations in the environment, and a ring beacon scanning device with 16 receivers is used to receive the infrared signals. The scanner is mounted on the robot to measure the relative angle between the robot and the beacon. The position of the mobile robot is estimated by fusing orientation information with motion controller commands or an odometer through an extended Kalman filter. Infrared sensors can be used to track targets when the transmitter and receiver are mounted on different robots. Tzuu-Hseng S. Li et al. [39] have designed a tracking control scheme for an autonomous mobile robot by using infrared sensors. The scheme involves a target mobile robot with an infrared reflector and a tracking mobile robot with an infrared receiver and a reflective sensor. The latter was designed to track the target robot. Juang J. G. et al. [40] designed wheeled mobile robots with integrated ultrasonic and infrared sensors that can move along walls and avoid obstacles. Ultrasonic and infrared sensors were used to detect obstacles and identify unknown environments. Infrared-based localization technology is currently mature. However, it is difficult for it to penetrate objects and is susceptible to its surroundings. Additionally, obstacles can directly block infrared rays in the infrared beacon-based localization method. As a result, the application of infrared sensors in mobile robot localization is limited.

In obstacle detection, infrared sensors are frequently used because of their rapid response and compact size [41,42,43]. Jianwei Zhao et al. [44] designed a multi-sensor mobile robot that uses infrared sensors mounted in front of the two front wheels at the bottom of the robot to detect ground pits.

### 2.4. LiDAR

LiDAR (Light Detection and Ranging) uses laser beams to measure the position and distance of target objects. Multiple laser beams are sent out from transmitters, and when they hit objects, they reflect back. The reflected beams are received by the LiDAR receivers. LiDAR measures the distance traveled by each beam using time-of-flight (ToF) and combines the distance information from all the laser beams to obtain information about the surroundings. LiDAR operates similarly to radar, but with a higher accuracy, measurement speed, and measurement distance compared to conventional radar.

In indoor mobile robot navigation, mobile robots mainly use 2D LIDAR to sense environmental information, while 3D LIDAR is more often used in the field of intelligent driving because it needs more information to process [45]. LiDAR is widely used for mobile robot localization due to its excellent distance measurement accuracy. Localization with LiDAR is based on an environment map [46]. Dirk Hähnel et al. [47] presented an algorithm for acquiring 3D models with mobile robots. Xipeng Wang et al. [48] devised a method for global localization using only schematic floor plans as prior maps. The method achieves global localization by matching features observed from LiDAR with features in the floor plan. The method performs comparably to a baseline system using a conventional LiDAR-based prior map. Haofei Kuang et al. [49] proposed a global localization method for mobile robots using 2D LiDAR. In this method, the neural occupancy field uses a neural network to implicitly represent the scene, and 2D LiDAR scans of arbitrary robot poses are synthesized by using a pre-trained network. The MCL system integrates the similarity between the synthesized scans and the actual scans as an observation model to perform accurate localization based on the implicit representation.

As LiDAR distance measurement is based on the principle of light reflection, the measurement has a large error in environments with glass obstacles or mirror obstacles. To overcome the limitations, J. Kim et al. [50] proposed a method for LIDAR-based localization schemes in glass wall environments. In this method, all candidate distances from the glass wall are pre-calculated based on the occupancy grid map and the reflection characteristics of the laser beam. The type of reflection phenomenon on the glass surface is then estimated on the basis of actual measurements. The robot’s local position tracking is performed by using a scan-matching method that considers the estimated results. Additionally, the fusion of ultrasonic sensors and LiDAR during obstacle detection can achieve a higher detection accuracy as ultrasonic sensors are not affected by glass or mirror obstacles.

In the absence of a prior environment map, LiDAR SLAM uses sensors to measure the surrounding environment and obtain a LiDAR point cloud, which is then used to estimate its position and construct a map based on the position information.

Typical 2D LiDAR SLAM methods include Gmapping [51], Hector SLAM [52], Karto SLAM [53], and Cartographer [54]. The Gmapping algorithm framework is based on the RBPF (Rao-Blackwellized Particle Filters) algorithm, and Gmapping makes two main improvements, including improving proposal distribution and selectively resampling. The Hector SLAM algorithm framework is based on the EKF (extended Kalman filter) algorithm. Hector SLAM can be used without an odometer and is suitable for airborne or uneven road environments, but it is prone to drift errors when the LiDAR is rotating too fast. Karto SLAM is the first open-source algorithm based on the PGO (pose graph optimization) algorithm. It relies on efficient linear matrix construction and sparse non-iterative Cholesky decomposition to efficiently represent and solve large sparse pose graphs. The most important point of Karto SLAM is the introduction of back-end optimization and loop closure detection. Compared with Gmapping and Hector SLAM, Karto SLAM is more advantageous for mapping in large environments. Cartographer is based on the PGO (pose graph optimization) algorithm. It eliminates the cumulative errors generated during the map building process mainly through loop closure detection, in which the submap is the basic unit. It accelerates loop closure detection by combining local and global data and has high real-time performance, which makes it a reliable 2D LIDAR SLAM method.

In recent years, many improvements have been made based on these SLAM methods. Xiang Y. et al. [55] designed a LIDAR-based localization and mapping method for indoor mobile robots. The method makes improvements in distribution and limiting the number of resamplings based on the original RBPF-SLAM algorithm. Han Wang et al. [56] proposed an intensity-assisted SLAM framework for LiDAR-based localization systems. The proposed SLAM includes intensity-based front-end odometry estimation and intensity-based back-end optimization. Yingzhong Tian et al. [57] proposed a method to improve the traditional ICP method by incorporating intensity into the calculation. This method reduces the number of iterations and arithmetic required. The method uses an objective function that considers the distance and intensity residuals of the LiDAR point cloud to determine the optimal initial transformation estimation. Weiwei Hu et al. [58] proposed keyframe extraction by clustering 2D LiDAR point clouds in indoor environments. In this method, firstly, the dimension of the scan is reduced to a histogram, and the key frames are extracted using the histogram. Then, the laser points in the key frames are divided into different regions using a region-segmentation method. Next, the points are clustered in separate regions, and the point sets from neighboring regions are merged. Finally, the sets with laser points below a threshold are discarded as anomalous clusters. Saike Jiang et al. [59] designed an autonomous navigation method for indoor mobile robots by fusing LiDAR, IMU, and encoder data. The method optimizes and combines the 3D point cloud information from multi-line LiDAR into a 2D LiDAR point cloud. This reduces computational power consumption in the SLAM process and improves map building efficiency. Additionally, this method can recognize obstacles at varying heights, overcoming the limitations of 2D LiDAR in acquiring height-related information and enhancing the safety of mobile robot movement. Weipeng Guan et al. [60] proposed a loosely coupled multi-sensor fusion method for VLP and LiDAR SLAM. In this method, the LiDAR sensor detects the surroundings to avoid obstacles during navigation and compensates for the cumulative error of the odometer. And VLP is used to provide high-precision pose initialization and correction for the LiDAR SLAM and odometer.

For obstacle detection, compared to ultrasonic sensors, LiDAR has significant advantages in distance measurement accuracy and efficiency for obstacles. J. H. Lee et al. [61] proposed a method for tracking multiple walking humans based on 2D LiDAR. The algorithm considers the fact that 2D LiDAR is often positioned at the height of human legs and uses the geometric characteristics of the legs to detect their position. The method utilizes a pendulum model of the angle between the two legs as well as an extended Kalman filter to extract the frequency and phase of the walking motion. Mozos O. M. et al. [62] used a multilayer 2D LiDAR for person detection. The multilayer LiDAR method scans different parts of the human body using different layers. A supervised learning approach is used to obtain a classifier for each layer of LiDAR data, which is then used to detect and recognize specific body parts. The results of each classifier are ultimately combined to achieve human body detection. Ángel Manuel Guerrero-Higueras et al. [63] devised a 2D LiDAR-based method for tracking people. The 2D LiDAR is mounted at knee height in a mobile robot, and the method is based on an offline trained full Convolutional Neural Network that can track pairs of legs in the environment. Yan, Z. et al. [64] proposed an online learning framework based on 3D LiDAR scanning for human detection. Ailing Zou et al. [65] proposed a nearest neighbor clustering algorithm based on the adaptive threshold to detect obstacles in the path of indoor robots by using 2D LiDAR.

### 2.5. Vision-Based Sensor

Visual sensors are devices that mimic the function of the human visual system. They capture, perceive, and interpret visible light information in the environment and convert it into digital signals or other forms of output for analysis, processing, and decision-making by robots or other systems. Compared to other sensors, visual sensors have the ability to represent the surrounding environment in the form of an image, making them capable of describing complex texture features in the environment. Moreover, vision sensors are widely utilized in the field of mobile robotics due to their relatively low cost. The main types of vision sensors are monocular cameras, binocular cameras, RGBD cameras [66], and event cameras [13].

The imaging principle of a monocular camera is similar to that of a normal camera and is usually represented by the pinhole camera model. Light enters through the camera’s aperture and is projected onto an imaging plane, typically a CMOS or CCD sensor [67]. The projected image is de-distorted and aligned with pixels to present a two-dimensional image similar to what is observed by the human eye. Despite its low cost, there are still significant technical challenges in research and development due to the difficulty of directly computing depth information. A stereo camera is a camera sensing system consisting of two monocular cameras, similar to the structure of the human eye. The depth information can be calculated from the baseline distance between the optical centers of the two cameras [68]. Depth estimation of a pixel point can be achieved by the underlying geometric model, as shown in Equation (1).

(1) $$z=\frac{fb}{d}$$

where *z* is the actual depth estimation, *f* is the camera focal length, *b* is the actual baseline distance, and *d* is the parallax between the pixel points in the left and right screen projections. From this equation, it can be seen that, due to the influence of the baseline distance b, a truncation error will occur if the depth z is too large, resulting in inaccurate depth estimation.

RGBD cameras are sensors obtained by integrating a depth sensor with a monocular sensor. The depth camera actively provides depth measurements at each pixel. Currently, the main depth sensors for RGBD cameras are infrared structured-light sensors and ToF (time-of-flight) sensors [69]. The main difference between the two cameras is the depth estimation principle. The infrared structured-light sensor calculates the depth information by recognizing the structured light pattern. The Time of Flight (ToF) sensor calculates the depth information of each pixel point in the camera by sending pulses of light and measuring the time it takes for the light to return. The depth map is then mapped to the RGB image using the relative position relationship between the sensors, resulting in a composite image of color and depth. However, due to the limitations of the depth sensor, searching for depth information is not feasible in large-scale and large-range spatial environments.

Event cameras are biologically inspired to work, so their imaging principles are not the same as those cameras described above. In traditional cameras, the main camera unit is a monocular camera, where the sensor captures the surrounding image at a constant frame rate to obtain environmental information. However, considering the frame rate and aperture factors, it will appear shadowy or blurred when facing environmental factors such as high dynamic and strong exposure. The event camera changes the recording method of the captured image to the recording of pixel changes. This means that the change in the pixels on the screen is recorded as an “event”, and the environmental information is read by the change in pixel brightness in the image on the output screen. As a result, the event camera can calculate quickly, with a frame rate of up to 1 KHz, and will not overexpose in high light intensity environments.

The light field camera is based on a monocular camera. A microlens array is added to the monocular camera to achieve a composite collection of environmental information. Due to the addition of the microlens array, the light sensor is able to sense light information in different directions at the same pixel point. The light field can be solved by the light information in different directions to achieve depth estimation in the surrounding environment. Similar to the binocular camera, it is able to estimate the depth of the surroundings from the line of sight in one frame. However, in contrast to the binocular camera’s simple two-frame camera, the light field camera can provide richer depth information for depth estimation or scene reconstruction at a later stage. The table below (Table 1) shows the pros and cons of each visual sensor.

In indoor environments, the effects of lighting, dynamic objects, and unstructured scenes can cause images that do not accurately represent the indoor environment. Additionally, before performing localization, the screen must be depicted as a scene that can be modified by the mobile robot. Therefore, it is necessary to pre-process the images to facilitate the extraction of environmental information by the camera.

Image pre-processing is mainly divided into the following aspects:

1: Eliminating image distortion.
2: Assigning semantic labels to specific objects. The extraction of specific objects or features in the picture can be achieved by neural networks or traditional feature extraction algorithms. Assigning semantic labels to them is more suitable for human understanding of environmental features.
3: Image Partial Reconstruction. In indoor environments, deep learning techniques like generative adversarial networks (GAN) and diffusion should be considered for reconstructing specific images due to the significant impact of light and dynamic objects on environmental representation. This reduces or reconstructs interfering factors in the image for the map construction process, such as dynamic objects, visible light, and shadows from occlusions. The goal is to acquire suitable images for scene localization.

Liu et al. [75] solved the issue of depth estimation between day and night by using the DCNN neural network. Considering the similarities and differences of the images between the environment with and without lighting, the images are divided into a private domain (lighting conditions) and a public domain (texture features). The reconstruction of a night image from a day image is achieved by the GAN neural network. This reduces the effect of the day/night boundary on the position and image construction errors. Bescos et al. [76] utilized CNN neural networks to implement multi-category semantic segmentation for the removal of dynamic objects and their accompanying shadows. GANs were employed along with a loss function based on image steganalysis techniques to achieve an absolutely static environment in the scene.

The conventional method for localizing mobile robots using visual sensors involves capturing image information with a camera and then comparing the images to determine the robot’s current position [77]. It is mainly divided into two major steps: feature extraction and data association. The decision of whether to extract features is divided between the feature point method and the direct method. For the data association part, the main goal is to achieve a real-time estimation of the camera’s own position change through the image comparison between frames. As the monocular camera cannot detect depth information directly, the relative positional relationship between the front and back frames of the camera corresponds to the rotation and translation in 3D space, which is achieved by establishing the rotational position between the front and back frames. That is, the pair of pole geometry problem, as shown in the following equation:

(2) $$\begin{array}{c} E=t^{\wedge }R \end{array}\;\begin{array}{c} x_{2}^{T}Ex_{1}=0 \end{array}\;\begin{array}{c} E=\left[\begin{array}{ccc} e_{1} & e_{2} & e_{3}\\ e_{4} & e_{5} & e_{6}\\ e_{7} & e_{8} & e_{9} \end{array}\right] \end{array}$$

where *E* is the essential matrix, which represents the positional transformation of the robot’s translation and rotation, and $x_{1}$ and $x_{2}$ denote the normalized coordinates of the corresponding pixel points of both on the projection plane. Since $e_{9}$ can be directly designated as one, it is possible to solve the polar geometry problem for eight pairs of pixel points corresponding to the eight pairs in the frame, thus realizing the position estimation of the camera. The depth information of the environment in the camera positioning scene is solved by triangulation, that is, the positional transformation relationship between the two frames and the corresponding pixel projection coordinates in the two frames are known, so as to calculate the corresponding depth information of the pixel points. This can be shown in the following equation:

(3) $$s_{1}{x_{1}}^{\wedge }x_{1}=0=s_{2}{x_{1}}^{\wedge }Rx_{2}+{x_{1}}^{\wedge }t$$

Although the monocular camera can realize the position estimation of the robot from eight mutually matching pixel points, it still has problems such as the large initialization error and degradation of position estimation due to pure rotation. For the stereo camera and depth camera, they can directly glean the pixel information from the environment and its corresponding depth information. Therefore, the position calculation is relatively simple, i.e., through the PNP algorithm [78]. As shown in the following equation:

(4) $$s\left[\begin{array}{c} u\\ v\\ 1 \end{array}\right]=\left[\begin{array}{cccc} t_{1} & t_{2} & t_{3} & t_{4}\\ t_{5} & t_{6} & t_{7} & t_{8}\\ t_{9} & t_{10} & t_{11} & t_{12} \end{array}\right]\left[\begin{array}{c} X\\ Y\\ Z\\ 1 \end{array}\right]$$

where *u*, *v* are the pixel points in normalized coordinates and *X*, *Y*, *Z* are the 3D coordinates corresponding to the pixel point.The *T* matrix represents the camera’s positional transformation. From the above, it can be concluded that the positional estimation of the camera can be achieved by six pairs of well-matched pixel points. Due to the addition of depth information, other constraints can be added to the PNP algorithm to achieve fewer points for the pose estimation, such as the P3P algorithm, the EPNP algorithm, etc., [68]. The event camera serves as the basic unit of the camera. Unlike monocular camera processing, the event camera performs feature matching by matching time, pixel coordinates, and light intensity changes in the event stream to generate environmental features in the frame that can be correlated with the data [79].

The position estimation of mobile robots can be implemented using a neural implicit approach due to the excellent generalization and iterative capabilities of neural networks. The traditional explicit representation of position estimation is to represent the relevant objects in the frame and the relevant features as the exact six-degree-of-freedom relevant point, line, and surface features. Neural implicit methods favor the representation of the features in the frame over functions or neural networks, such as voxels, meshes, distance fields, etc. The advantage of implicit representation is that it can effectively represent complex texture features and more effectively represent complex surfaces such as voids and surfaces as opposed to explicit point clouds or grids. Furthermore, neural implicit methods have the advantage of compact spatial representation and the ability to store a large amount of information, allowing them to represent a larger map space with less memory compared to explicit networks. By using neural networks to predict and update maps, neural implicit methods have end-to-end learning capabilities that allow for greater flexibility in updating maps and estimating poses in real-time applications. Compared to traditional feature extraction and matching-based methods, neural implicit methods have better adaptability and robustness when dealing with dynamic scenes or fast movements. Zhu et al. [80] employs a neural implicit representation to capture the geometric and appearance attributes of a scene in terms of hierarchical network features. They utilize a pre-trained neural implicit decoder encoded at different spatial resolutions. By rendering the generated depth and colour images, they optimise the feature mesh within the field of view cone by minimising the re-rendering loss function.

There are many types of visual map building, but map functions generally have the following characteristics:

1: The ability to effectively represent the characteristics of the surrounding environment.
2: The amount of computation and storage required can match the hardware carried by the robot.
3: The ability to achieve the functionality required by the mobile robot.

As for map types, the main ones are point cloud maps [81,82], 3D occupancy grid maps [83], TSDF maps [84], semantic maps [85], etc.

A point cloud map generally consists of a camera frame that represents the camera’s position, the camera’s historical trajectory, and a pixel point cloud. It accurately depicts the surrounding environment and the camera’s position transformation. However, the redundant point cloud features result in large storage space and construction costs. Vijayanarasimhan et al. [86] introduced the SfM-Net network, which retrieves depth maps and performs camera pose estimation using a single video stream. The network generates a corresponding depth image from the input single image and fuses the depth point cloud to create a comprehensive representation. Finally, the positional relationship between the resulting images is computed based on the input pair of consecutive frames. Furthermore, the method identifies and segments any moving objects within the scene, presenting them in the form of a mask.

The 3D occupancy grid map divides space into occupied subspaces based on a certain volume. If an object is present in the occupied subspace, it is considered occupied. While it sacrifices clarity in environmental information representation compared to the point cloud map, it significantly reduces computation and map information storage requirements. This is advantageous for constructing large-scale maps. Cao et al. [87] proposed a 3D semantic occupancy raster map completion method for inferring indoor and outdoor scenes in a single RGB image. The method is applicable to a wide range of scenes and environments and does not require additional depth sensors or specific scene types. The article introduces the FLoSP mechanism to correlate 2D and 3D networks. Additionally, a new loss function is proposed to optimize inference results by taking into account the semantic relationships and local structure of voxel groups. This improves the quality and consistency of scene completion.

TSDF maps are maps represented by the TSDF notation (Truncated Signed Distance Function). Similar to three-dimensional occupancy grid maps, TSDF divides the space equally into an infinite number of voxel representations denoted by (x, y, z, v). The map is iteratively updated by a mobile robot in a continuous positioning process. Since updating the voxels directly is required, RGBD cameras are generally used to implement TSDF map construction. Kim et al. [88] predicted 3D Truncated Signed Distance Function (TSDF) voxels from a single RGB image by integrating depth prediction and TSDF generation processes seamlessly. This approach enables the generation of highly accurate TSDF maps with greater efficiency compared to TSDF conversion from depth prediction alone. The comprehensive TSDF model is utilized to improve the precision and robustness of camera pose estimation, resulting in more effective scene facilitation.

Semantic maps differ from the above three maps. They are created by using image processing or deep learning network methods to extract the physical properties of points, lines, surfaces, or object features from an image and assign corresponding semantic information during the map construction process. Thus, we can create maps that align with human thinking and are more conducive to human–machine interactions based on the aforementioned maps. Yu et al. [89] proposed a methodology for extracting semantic segmentation information from the conventional SLAM framework. This process is executed as an independent thread within the image preprocessing module. The approach effectively identifies and eliminates dynamic objects present in each frame by combining semantic data with the concurrent detection of motion feature points. A dense semantic octree map is generated to meet the specific task requirements associated with advanced robot decision-making and path planning.

The development of mapless navigation can improve the computational speed of indoor mobile robots, which is conducive to autonomous decision making and path planning in dynamic environments. However, how to set up an effective reward mechanism and network structure is a major research challenge in mapless navigation. Kulhanek et al. [90] used auxiliary tasks consisting of reward prediction, pixel control, and semantic segmentation prediction to achieve effective training of neural net structure, and introduced LSTM as a memory module in the process of the network structure to cope with the before and after state correlation problem in the POMDP problem. Xiao et al. [91] addressed the problem of how to make a mobile robot learn to find the target point by passing through corners and such places when the initial target is not visible, creating arrival rewards, collision penalties, and proximity rewards. An “action consistency” mechanism was designed to achieve consistency between the learned behaviour of the mobile robot and its accumulated experience. Xie et al. [92] proposed a framework for a mapless navigation method for mobile robots based on a hierarchical approach with DL and DRL. It is assumed that the mobile robot can be provided with navigation instructions and images of key locations before task execution. The upper layer “watches” the navigation instructions and the current state to determine whether it is time to reach the place where it needs to act according to the navigation instructions. The lower layer is responsible for completing the navigation function of the mobile robot in between the upper layer’s updated decisions.

Vision cameras have a significant advantage in obstacle recognition compared to other sensors, making them a popular choice for obstacle avoidance in mobile robots. The obstacle avoidance system designed by Tai et al. [93] does not provide real-time estimations of the robot’s position during obstacle avoidance but builds a convolutional neural network for the original depth image. Subsequently, it establishes a fully connected network with five decisions on obstacle avoidance for the mobile robot, which enables the mobile robot to autonomously realize obstacle avoidance planning during indoor movement. Cristóforis et al. [94] achieved autonomous obstacle avoidance by building a “teach-learning” system that only requires texture information about the path of the screen rather than the position estimated since localization.

The perception of indoor mobile robots covers a variety of map representations. Point cloud maps are discrete point cloud data acquired by sensors such as LIDAR or camera sensors that provide highly accurate information about the structure of the environment. Three-dimensional occupancy raster maps divide the environment into a three-dimensional grid that is used to represent the location and occupancy of objects.TSDF maps provide highly accurate geometric information by modelling the surface of an object. Semantic maps further incorporate semantic information to enable the robot to understand the semantic meaning of different areas in the environment. In contrast to traditional navigation techniques, the mapless navigation function based on image information is a lightweight and efficient navigation method. It acquires environmental images through sensors such as cameras, and uses deep reinforcement learning and other methods to achieve end-to-end perception and decision making by learning the environmental features in the images, so as to realize autonomous navigation for robots. As the method does not need to pre-construct maps, it alleviates the dependence on expensive sensors, while showing a strong adaptability in dynamic environments. Therefore, mapless navigation based on image information is robust, with a small computation and memory footprint, making it perform well on resource-constrained mobile robot systems.

### 2.6. Radio Frequency Technologies

Radio frequency (RF)-based sensing technology is mostly used for indoor mobile robot localization. The RF-based localization system comprises beacon stations (BS) that transmit radio signals and mobile robots that receive them. Unlike infrared and ultrasound-based beacon location systems, RF waves can penetrate doors and walls, providing ubiquitous coverage of buildings. RF-based localization systems applied to indoor mobile robots typically include: WiFi [95], Bluetooth [96], Zigbee [97], Ultra-Wideband (UWB) [98], and Radio Frequency Identification (RFID). The advantages and disadvantages of the RF techniques [99] are shown in Table 2.

#### 2.6.1. Radio Frequency Technology Localization Algorithms

**Figure 6:**
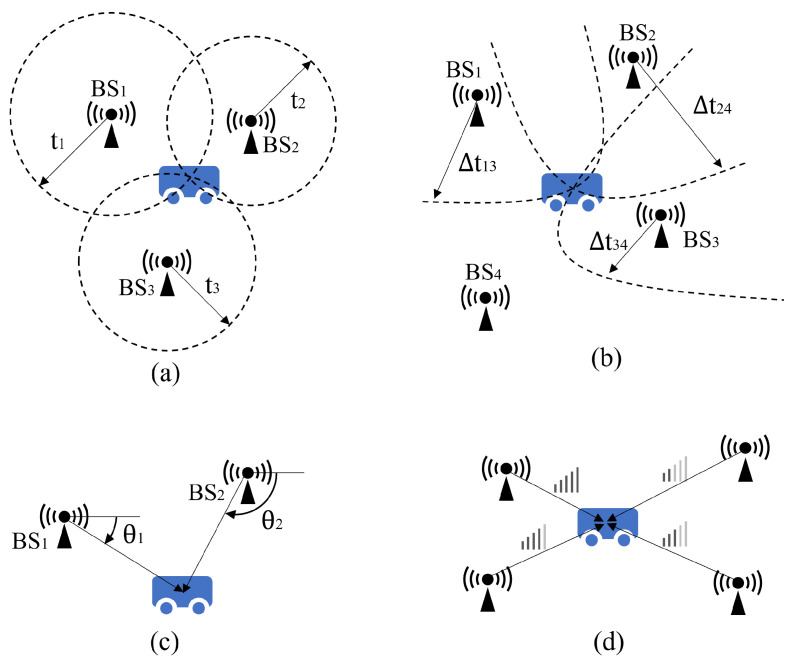
RF localization technologies: (**a**) Time of Arrival (TOA). (**b**) Time Difference of Arrival (TDOA). (**c**) Angle of Arrival (AOA). (**d**) Received Signal Strength Indication (RSSI).

RF-based indoor localization technologies use two main methods: geometry and fingerprinting. The geometry-based localization method determines the location of the mobile robot using geometric knowledge, either by measuring the distance to fixed beacon stations (BS) or by calculating the received signal angle. Several typical RF-based positioning algorithms are Time of Arrival (TOA), Time Difference of Arrival (TDOA), Angle of Arrival (AOA), and Received Signal Strength Indication (RSSI), as shown in Figure 6.

TOA is a localization method that measures signal propagation time. A signal sent from a transmitter on a mobile robot travels through the air at approximately the speed of light. When the signal reaches receivers mounted at multiple beacon stations (BSs), the receivers record their respective arrival times [100]. The distance of signal propagation is calculated by measuring the propagation time. This allows for the localization of the mobile robot using the triangulation method, as shown in Figure 6a. The signals emitted by the transmitters are time-stamped to calculate the propagation time. It is crucial to synchronize the time of the beacon stations with the time of the mobile robot [101].

TDOA is a method used to determine the location of a signal transmitter by measuring the time difference between the signal’s arrival at different receivers [102]. The arrival time of the same signal is recorded by multiple signal receivers, and the time differences between the different receivers are calculated. These time differences are then used to construct a system of equations. Each equation represents the positional constraint of the corresponding receiver on the transmitter. These constraints are reflected in the coordinate graph as hyperbolas. The position of the signal transmitter, i.e., the mobile robot, is determined by solving these equations, as shown in Figure 6b. Unlike the TOA method, the TDOA method only requires time synchronization between the beacon sites, not between them and the mobile robot [103].

AOA is a method used to determine the position of a signal transmitter by measuring the angle at which the signal arrives at different receivers [104]. The signal receiver consists of multiple signal-receiving elements, and the angle of arrival is calculated by comparing the arrival times or phase differences of the signals between the different receiving elements. The angle of arrival of a signal is recorded by a plurality of signal receivers. Geometric relationships are then used to calculate the position of the signal transmitter, which is the mobile robot, as shown in Figure 6c.

The received signal strength indicator (RSSI) is a measurement of the power of a radio signal that has been received. Signal strength decays during propagation, which is characterized by the propagation power loss model. Therefore, the strength of the received signal can also be used to calculate the signal propagation distance. Triangulation is then used to measure the position of the mobile robot [105].

Fingerprint localization is a technique used to determine location by comparing received signals with a pre-established fingerprint database [106]. It consists of fingerprint database construction and location estimation. The process involves deploying a set of RF sensor receiver stations throughout the area to collect RF signals at each sampling point and record their characteristic parameters. The sampling point locations are correlated with these characterization parameters to construct a fingerprint database. This database contains pre-measured and recorded correspondences between signal features and locations. The most commonly used signal characteristic parameter is RSSI. Once the fingerprint database is established, the RF sensor stations collect real-time wireless signal parameters, which are then matched and compared with the fingerprint information in the database [107]. Accurate target location is estimated through the use of comparison and inference algorithms. Compared to the geometry-based method, the fingerprint localization method is more accurate. However, constructing the fingerprint database requires significant effort, and it must be re-collected and constructed if the signal receiver station’s location changes [103].

#### 2.6.2. WiFi

WiFi is a widely used wireless technology that follows the IEEE 802.11 standards. WiFi utilizes wireless communication frequencies of 2.4 GHz and 5 GHz. It can support multiple device connections simultaneously and is widely used in indoor settings such as factories and offices. While WiFi technology offers advantages such as convenience, flexibility, and high speed, it has limitations when used for indoor localization. Factors such as the volatility of the WiFi signal and the non-line-of-sight (NLOS) path may affect the accuracy of localization.

For WiFi-based positioning methods, RSSI is most commonly used because of its high accuracy. Hemin Ye et al. [108] optimized the traditional WiFi fingerprint positioning method. Compared to traditional methods for fingerprint localization, this approach reduces the distance between sampling points and improves fingerprint matching accuracy by collecting and normalizing WiFi signals from different time periods. Additionally, the method uses Mahalanobis distance as a similarity reference and filters out noise using an improved adaptive K-value WKNN algorithm to enhance location estimation accuracy. Zhang L. et al. [109] optimized WiFi-based RSSI localization with Deep Fuzzy Forest. In their work, a deep fuzzy random forest is used as a mapping function, which estimates the corresponding coordinates of fingerprint vectors from an offline database. The deep fuzzy forest can inherit the merits of decision trees and deep neural networks within an end-to-end trainable architecture. Guangbing Zhou et al. [110] combine WiFi, vision, and LIDAR for the indoor localization of mobile robots.The WiFi-based RSSI fingerprinting localization method was used for coarse area estimation.

#### 2.6.3. Zigbee

Zigbee is a wireless communication technology based on the IEEE 802.15.4 standard. It uses a distributed network topology that supports multiple devices communicating with each other, with all terminators communicating directly with the coordinator. ZigBee has a low cost, low power consumption, and low data transfer rate compared to WiFi standards, as well as a longer latency time.

Zhang Lei et al. [111] conducted an experimental analysis of Zigbee location using RSSI technology for the influence of reference node layout and number on the location accuracy. The experimental results indicate that Zigbee reference nodes with triangular layouts achieve higher localization accuracy than those with rectangular and prototypical layouts. Anbalagan Loganathan et al. [97] combined Zigbee-based RSSI and odometry for the indoor localization of mobile robots. Their method fuses the results of Zigbee-based RSSI triangulation with odometer data through an adaptive filtering method to find the robot’s position. Zhu Wang et al. [112] designed a Zigbee-based localization algorithm for mobile robots. The method corrects the RSSI ranging results by least squares and uses triangulation to calculate the robot’s position. The coordination nodes and mobile nodes of the ZigBee network are embodied in mobile robots, thus enhancing the mobility and flexibility of the network.

#### 2.6.4. Bluetooth

Bluetooth is a standardized wireless communication technology based on the IEEE 802.15 standard. It is commonly used for short-range data transmission due to its low power consumption, low cost, high security, and ease of use. Bluetooth technology is widely used in various electronic products, automobiles, and medical devices. However, the accuracy of indoor positioning is affected by the non-line-of-sight (NLOS) path due to obstacles affecting Bluetooth signal propagation.

Aswin N. Raghavan et al. [113] proposed a method for the localization of mobile robots using Bluetooth. This method is based on RSSI for distance estimation and uses trilateration measurements for localization. The localization accuracy is within 1 m. Y. Yamami et al. [114] designed a Bluetooth-based AOA method for indoor mobile robot localization. The method makes use of Bluetooth 5.1 with the Constant Tone Extension (CTE) at the end of a packet, enabling the Bluetooth module to receive signals from multiple antennas simultaneously. In the study conducted by Katrina Weinmann et al. [115], a mobile robot controller employs Bluetooth as a sensor to measure the localization of the target object. The Bluetooth beacon is mounted on the target object, while the mobile robot is equipped with a Bluetooth antenna array. The mobile robot achieves target tracking by estimating the direction and distance of the target object. The direction is estimated using the AoA method, while the distance is estimated using RSSI. Astafiev Alexandr et al. [116] experimentally analyzed indoor localization based on a sensor network using Bluetooth Low Energy (BLE) beacons. The experiments demonstrate that the transmission of BLE signals can be affected by the presence of other radio signals in the localization area. The level of signal interference directly impacts BLE-based localization accuracy, resulting in greater errors and a lower accuracy. Additionally, obstacles such as people interfere with the RSSI measurement of BLE, further affecting positioning accuracy. To enhance the anti-interference capability of the BLE positioning system, it can be combined with other sensors, such as the IMU.

#### 2.6.5. Ultra-Wideband (UWB)

UWB (Ultra-Wideband) technology is a wireless communication technology that uses short, non-continuous pulse signals to transmit data. It has a wide frequency bandwidth and offers advantages such as low power consumption, fast transmission rate, and strong anti-interference capability. Additionally, UWB is theoretically less affected by non-line-of-sight (NLOS) paths, making it highly accurate and stable for indoor localization.

Dongqing Shi et al. [117] designed a positioning system based on UWB sensors. To optimize localization results in the presence of nonlinearity and noise in UWB ranging, the system employs the gradient descent method and the least squares method. Jiajun Leng et al. [118] designed a two-stage UWB positioning algorithm for indoor mobile robots. First, the distance measured by the TDOA method was optimized by Gaussian filtering. Then, the final location coordinates were obtained by weighting the corrected range values with coordinates.

To reduce the influence of the LOS/NLOS environment on positioning accuracy, Peisen LI et al. [119] designed a positioning method combining the inertial navigation system (INS) with Ultra-Wideband (UWB). In this method, an interactive multiple model (IMM) algorithm is proposed to integrate the different distance error characteristics of LOS and NLOS states. The algorithm uses two Kalman filter models for different states and transforms them using the Markov chain. The filter results are then fused using weighting for position estimation. The localization method corrects the UWB’s estimated position with the INS position estimation results, resulting in the final localization estimation. Tao Xu et al. [120] proposed a Weighted Adaptive Kalman Filter (WAKF) positioning method based on UWB andodometry. To minimize the effect of LOS/NLOS scenarios on UWB positioning accuracy, a Kalman filter is used to integrate the odometry data. Additionally, the method uses power differences to distinguish between LOS and NLOS environments, and it adjusts the Kalman filter weights accordingly. Haoyu Zhou et al. [121] designed an online multi-robot SLAM system based on Lidar/UWB fusion. The system fuses distance measurements provided by UWB sensors with Lidar data provided by different mobile robots and constructs a globally consistent map based on the UWB coordinate system.

#### 2.6.6. Radio Frequency Identification (RFID)

RFID (Radio Frequency Identification) technology uses radio waves for data transmission and identification. It typically consists of three components: a tag, a reader, and a data processing system. RFID tags are small devices that contain a chip and an antenna and are used for data storage and transmission. The reader communicates with the tag via radio and reads or writes the tag’s information. The RFID data are processed and managed by the data processing system. RFID technology offers the benefits of low energy consumption and low system costs. However, it has a short signal transmission distance of about 1–5 m, which requires the presetting of numerous beacon sites for large-area localization. RFID is advantageous for short-range target tracking and identification.

Haibing Wu et al. [122] proposed a method for mobile robot navigation based on RFID technology to track targets. The method uses a particle filter to measure the real-time relative position between the mobile robot and the RFID-tagged object based on the RFID phase difference observation model. Jun Wang et al. [123] proposed a SLAM method for mobile robots based on high-frequency band RFID by using the particle smoother for landmark mapping and the particle filter for the self-localization of the mobile robot. F. Shamsfakhr et al. [124] proposed a mobile robot localization method based on passive UHF radio frequency identification technology. The method combines odometry and RSSI-based position estimation results to determine the robot pose by the linear least squares method.

### 2.7. Summary and Discussion

IMU sensors have a high output frequency and are extensively adopted in mobile robot localization and SLAM fields. As a proprioceptive sensor, it is not influenced by the environment, but it does not have a role in obstacle detection. To mitigate error accumulation, an IMU is often used in conjunction with encoders as a short-term odometer and is part of the sensor fusion method in mobile robot positioning and SLAM. Ultrasonic sensors are less accurate in distance detection than infrared sensors and LiDAR. Therefore, they are rarely used as primary sensors in mobile robot localization and SLAM in recent studies. However, they have significant advantages over other sensors in obstacle detection and are widely used in mobile robot obstacle avoidance. Infrared sensors, as optical sensors, are susceptible to other light sources or reflections in the environment. However, infrared sensors are very low cost and are often used as auxiliary sensors in mobile robots, responsible for obstacle or target distance detection. LiDAR and vision sensors are the most commonly used sensors in SLAM for mobile robots, and they can both be used for navigation in unknown environments. In contrast, LiDAR has a higher distance measurement accuracy, and vision sensors can collect richer information about the working scenes. However, the accuracy of LiDAR measurements can be affected by transparent or reflective objects in the environment. Vision sensors are advantageous for detecting obstacles or targets due to their excellent object recognition technology. They can efficiently filter out obstacle data, improving localization precision during robot navigation. Radio frequency (RF) sensors are widely adopted in mobile robot localization, and the localization accuracy will not be influenced by environmental factors such as low-visibility conditions. However, RF sensor-based localization requires additional anchor nodes with prior position information and cannot provide orientation information for mobile robots. A comparison of results of the relating sensors is demonstrated in Table 3.

## 3. Overview of Multi-Sensor Fusion Sensing Technologies

Various sensors can gather diverse forms of measurement data. Sensor fusion allows for a more comprehensive acquisition of sensory information, thereby enhancing localization efficiency in task completion. It is important to note that each sensor has its own superiorities, limitations, and potential scenarios. It is noted that sensor information fusion is able to enhance mobile robot positioning accuracy, obstacle recognition efficiency, and other characteristics by leveraging strengths and compensating for weaknesses. This approach can meet the requirements of diverse working environments. For instance, visual sensors provide a vast amount of image data that is valuable for recurrent closed-loop detection and optimization of the same scene in large-scale environments. This method enables the elimination of cumulative sensor errors. However, visual sensors in indoor environments are also limited by their image information. They suffer from long image processing times (generally 30 fps is required for the vision SLAM image in real-time), and environmental information is susceptible to occlusion. Therefore, improving visual sensor performance through other sensors is also a hot topic for mobile robot perception. As for multi-sensors, sensors that are often mixed with visual sensors are sonar [125,126], laser rangefinders [127,128], radio frequency identification (RFID) [128,129], inertial sensors [130,131], GPS [132,133]. However, in the case of GPS sensors, they are based on satellite systems to achieve global positioning. In indoor environments, objects are affected by buildings as well as other signals, causing a significant reduction in their positioning accuracy, implying that GPS may be inappropriate for mobile robot location positioning in indoor obscured environments [134,135].

Sensor data fusion algorithms mainly include improved algorithms based on Kalman filters, particle filters, and neural networks.

### 3.1. Multi-Sensor Fusion Algorithm Based on Kalman Filter

**Figure 7:**
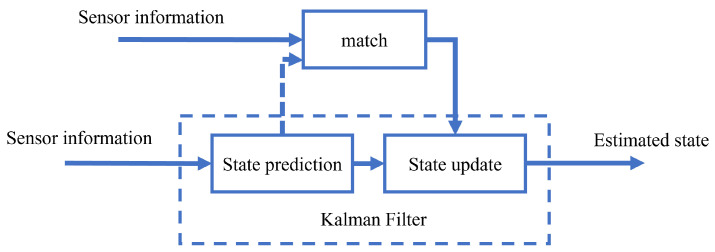
Multi-sensor fusion algorithm based on Kalman filter.

Kalman filtering methods are mainly used for fusing high-frequency and high-precision dynamic data and have been widely applied in multi-sensor fusion [51,136,137]. When assuming that the system state model and observation model are linear and follow a Gaussian distribution and that the noise is also Gaussian, the Kalman filtering method only requires the mean and variance of the noise to iteratively solve for the state. Kalman filter mainly includes prediction and update steps to realize real-time state estimation of the mobile robot from the previous frame to the current one, as shown in Figure 7. However, the traditional Kalman filter cannot meet the requirements of nonlinear problems. Therefore, researchers have developed improved versions of the Kalman filter, such as the Extended Kalman Filter method (EKF) and the Untraceable Kalman Filter method (UKF), to deal with nonlinear problems.

Daniel Magree et al. [138] proposed a modified navigation system by integrating visual SLAM and LiDAR SLAM, thus achieving an EKF-based inertial navigation system. In this vision SLAM approach, the robot’s position can be determined by analyzing the environment’s image features. The position is estimated and updated using the EKF method. LiDAR SLAM is based on the Monte Carlo method for scan matching. Its results are used to correct the results of visual SLAM and reduce the effect of ambiguous geometry on visual SLAM. Chengguo Zong et al. [139] presented an EKF multi-sensor information fusion method for obstacle avoidance in mobile robots. This method utilizes the previous moment’s pose and encoder data to estimate the predicted position. The observed position can be obtained on the basis of observations from ultrasonic sensors, infrared sensors, and an electronic compass. The EKF updates the pose estimation by incorporating the predicted and observed positions, resulting in the optimal estimate of the required pose. LILI MU et al. [140] offered a SLAM approach that fuses LIDAR, an RGBD camera, an encoder, and an IMU. This method utilizes UKF to fuse the data from the four sensors for localization. In this method, the current position of the mobile robot is obtained from the IMU and wheel encoder, and it is input into the UKF for updating the rough position, which is used as the initial position for scanning matching by LIDAR and depth camera point clouds. This speeds up the matching speed of the point cloud and improves the matching accuracy. The optimized pose obtained by point cloud matching is sent to UKF as the measured value. Ping Jiang et al. [141] designed a rank Kalman filtering method for obtaining the robot motion trajectory by utilizing the IMU and LiDAR observations. The proposed method improves the localization accuracy of indoor mobile robots under nonlinear and non-Gaussian noise models. Lin et al. [142] proposed an iterative Kalman filtering scheme by using the error states. It utilizes the IMU information as an information carrier to discretize the continuous motion model and integrate the three sensors to generate more accurate trajectories. Wei Xu et al. [143] proposed a LiDAR–inertial odometry framework that contains a state estimation module and a mapping one. In this way, the IMU and LiDAR data are input into the iterative Kalman filter for optimal estimating position.

### 3.2. Multi-Sensor Fusion Algorithm Based on Particle Filter

The Kalman filter and its improved algorithms are only capable of handling Gaussian distributions. When dealing with arbitrary distributions, the adoption of Kalman filter-related methods may result in more errors. The particle filter-based approach, known as the Monte Carlo algorithm, is able to deal with the arbitrary distribution of multiple samples [144]. In this method, the probability density function of the indoor AMR’s position can be approximated using a set of “samples” or “particles”, and regions with a larger number of particles have a higher probability. Each particle denotes a hypothetical pose of the considered mobile robot, while its weight indicates the extent of the match between the hypothesis and the true state. Typically, larger samples are used for global localization, and smaller samples are used for pose tracking.

**Figure 8:**
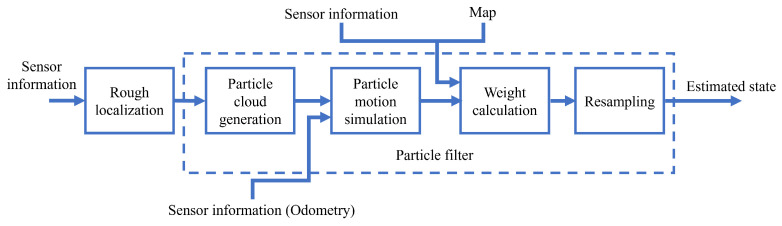
Multi-sensor fusion algorithm based on particle filter.

When using the Monte Carlo algorithm for global localization, particles must be distributed throughout the entire map due to the unknown initial position. However, this can result in a significant amount of computation and reduced localization efficiency. To enhance global localization efficiency, researchers have conducted numerous studies on sensor fusion. This involves rough localization followed by accurate localization, as shown in Figure 8. Song Xu et al. [145] designed a global localization method using the camera and LiDAR. In this method, global localization includes two processes: coarse localization and fine localization. In coarse localization, existing features from a pre-trained convolutional neural network (CNN) are used for determining the candidate positions based on images extracted from a monocular camera. In fine localization, the precise robot position is estimated by adaptive Monte Carlo localization based on LiDAR, where the candidate position is used as a seed for initial random sampling. Gengyu Ge et al. [146] proposed a method for localizing mobile robots by fusing camera and LiDAR data. The method first obtains coarse localization from the camera’s text-level semantic information, and then enhances the localization by using the Monte Carlo localization (MCL) method with LiDAR data. Huang Y et al. [147] presented a global localization approach using LiDAR and dual AprilTag for enhancing the global localization of mobile robots. This method utilizes AprilTag-based localization results as a rough localization for the adaptive Monte Carlo global localization algorithm. Based on this rough localization, precisely located particles are generated, which enhances the efficiency, as well as the success rate during global localization.

### 3.3. Multi-Sensor Fusion Algorithm Based on Neural Network

**Figure 9:**
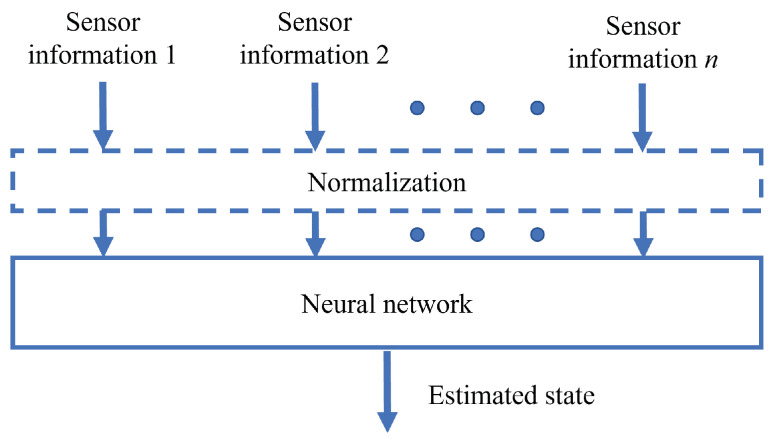
Multi-sensor fusion algorithm based on neural network.

In recent years, researchers have introduced machine learning technology to mobile robots, which has led to the rapid development of mobile robotic systems [148,149]. Neural networks have also been widely studied and applied in multi-sensor fusion for mobile robots. Neural network-based methods can automatically extract features from data, avoiding the difficulty and uncertainty of manually designing features while having an improved generalization ability and robustness, as shown in Figure 9. Compared to Kalman filter-based methods, neural network-based methods are more capable of handling nonlinear problems and achieving higher accuracy.

Carlos Eduardo Magrin et al. [150] proposed a hierarchical sensor fusion (HSF) method with an artificial neural network (ANN) for mobile robot self-localization. This method uses a two-level multilayer perceptron (MLP) to fuse the normalized sensor data of the sonar octagon, digital compass, and wireless network signal strength measure. Li et al. [151] applied neural network fusion of LiDAR and an encoder for mobile robot localization. In this method, LiDAR data and odometer data obtained through an encoder are fed into a three-layer neural network for fusion to improve the mobile robot’s positioning accuracy. Chi Li et al. [152] presented a deep learning-based approach to localizing a mobile robot using a 2D LiDAR and an IMU. The method utilizes a recursive convolutional neural network (RCNN) to fuse laser and inertial data and estimate the scan-to-scan attitude. The approach demonstrates greater robustness than traditional geometric-based methods in challenging situations, such as high angular speeds. Jieun Lee et al. [153] provided a deep neural network architecture (FusionLoc) for mobile robot relocalization with a camera and 2D LiDAR. This method contains two feature extraction modules, i.e., a multi-head self-attention (MHSA) module and a regression one. To be more specific, the feature extraction module extracts features from RGB images and LiDAR point clouds, respectively; the MHSA module performs feature fusion; and the regression module realizes position estimation. This approach is capable of achieving improved performance as compared with traditional end-to-end relocalization approaches that only acquire data from one sensor. A fingerprint-assisted localization scheme for mobile robots is explored, which fuses RSSI and magnetometer measurements [154]. However, the accuracy and stability of RSSI-based fingerprint localization methods can be affected by various environmental disturbances. The magnetic field strength can provide information about the level of disturbances. The method uses a fusion of magnetometer measurements and fingerprint data from RSSI for mobile robot localization through multilayer perceptron (MLP) feedforward neural networks. Andres J. Barreto-Cubero et al. [155] fused an ultrasonic sensor and a stereo camera with 2D LiDAR through the artificial neural network to improve obstacle detection in mobile robots by using the capability of ultrasonic sensors to detect glass and the ability to accurately detect objects in the 3D environment of the stereo camera.

### 3.4. Other Multi-Sensor Fusion Algorithms

The weighted average method is a direct and effective approach that simplifies the data fusion process during algorithm design and ensures real-time calculations. The core idea of the method is how to weight multiple sensors and how to determine the correction method. Wen et al. [156] combine an incremental encoder with a camera to calculate the encoder-based local position and globally update the position through the similarity of the camera to the environment. Jiang et al. [157] achieved the combination of feature point cloud maps and grid maps by establishing joint error co-visual between LiDAR and the monocular camera. Zheng et al. [81] divided the SLAM system into two subsystems, i.e., VIO and LIO. The coupled relationship between the two subsystems is achieved by sharing the point map data between the two to achieve accurate position estimation. However, because the weighted average method does not take into account the relationship between the front and rear states of the robot, its anti-disturbance ability is not excellent in the face of highly dynamic environments. Kosisochukwu Pal Nnoli et al. [43] fused infrared and ultrasonic sensors to detect obstacles. Their approach uses an error-filtering covariance and averaging algorithm to logically fuse distance measurements from a pair of infrared and ultrasonic sensors to track the proximity of environmental obstacles within a 180-degree range in front of a mobile robot.

Graph optimization algorithms in SLAM are also used as a framework for multi-sensor fusion. Ran Liu et al. [158] presented a map-building scheme for UWB, LiDAR, and odometry. In this method, a graph optimization framework with two optimization processes is designed to handle the fusion problem of UWB ranging information, odometry, and LiDAR information. The first optimization process takes the poses of the UWB nodes as vertices in the graph, and the edges of the UWB ranges and odometers are used as constraints, and roughly optimized robot trajectories are obtained. The second one integrates the edges determined based on LiDAR ICP scan matching to obtain the accurate robot trajectory. Along this line, then, the resulted trajectory can be incorporated into the LiDAR data, yielding the occupancy map of the industrial scenes. The graph optimization process can better deal with the influence of nonlinear systems on the robot. However, the algorithm’s high calculation complexity, caused by the need to traverse previous state variables, can make real-time updating difficult during robot navigation. The robot’s state is generally optimized when loop closure and relocalization are triggered.

## 4. Discussions and Future Trends

### 4.1. Discussions

The perception system of an AMR offers information about the robot’s association with the environment, which is essential for successful navigation. To ensure the indoor mobile robot completes the navigation task successfully, it is essential to reasonably select and optimize the sensors and algorithms used in the perception system based on the specific needs of the scenario.

IMU-based trajectory inference methods can only be used for short periods of time and can be used to assist other localization systems. For mobile robot localization, beacon-based positioning systems (including RF sensors, ultrasonic sensors, and infrared sensors) can provide stable and accurate absolute position information. However, they require regular installation and maintenance, and widespread deployment can lead to increased costs. LiDAR, or vision cameras, can be used for accurate and stable positioning. However, their localization relies on environmental information extracted by the sensors, making them susceptible to changes in the environment. For scenarios with frequent changes, beacon-based localization methods are more appropriate.

For the SLAM of a mobile robot, both LIDAR and vision cameras have their own advantages. Laser point cloud data require less computation, are easy to compute, are accurate in ranging, and are less susceptible to large changes in lighting. However, the environmental information collected by LiDAR is not sufficient, and the map constructed using 2D LiDAR only contains distance information about the environment. The rotating mechanism of the device is simple, which negatively impacts the stability of its internal structure over time. Vision SLAM provides a more detailed description of the environment, including texture information, which enhances accuracy and enables the recognition of differences between environments. While laser SLAM has limitations, vision SLAM, equipped with a camera, offers greater stability in terms of internal structure. Mobile robots with only a single camera as a sensor in indoor environments are limited by environmental conditions and lack robustness.

For obstacle detection, vision cameras are effective for recognizing obstacles as they provide rich environmental information. Ultrasonic sensors and LiDAR are precise in measuring the distance and position of obstacles.

### 4.2. Future Trends

Benefiting from computer science, artificial intelligence, and visual approaches, perception technology will develop rapidly, and the following aspects are worth emphasizing:

**1:**  **Multi-source Information Fusion.** The study and development of mobile robots have extended their possible applications, along with increased demands for their usage. However, a single sensor may struggle to satisfy the navigation requirements of complex scenes due to its inherent limitations. Along this line, then, multi-sensor fusion becomes a crucial area of research and development for indoor AMR. Multi-sensor fusion can improve the accuracy, efficiency, and stability of localization, map building, and obstacle detection by providing more comprehensive environmental information to the perception system. Multi-sensor fusion provides accurate and comprehensive information to the decision-making system, enabling mobile robots to adapt better to complex environments and complete navigation tasks more efficiently and robustly.
**2:**  **Flexible and efficient optimization strategies.** When mobile robots work in various complex and dynamic environments, there is a large amount of unknown model noise and roughness in the observation information obtained by sensors. To ensure the efficiency, stability, and accuracy of the AMR system, appropriate optimization strategies should be explored based on the specific environment and dynamic changes. In addition, there are many complex nonlinear operations in the optimization process. To improve computational efficiency when dealing with large-scale or multi-modal data, methods such as parallel computing and distributed computing can be adopted.
**3:**  **Integration with neural networks.** Since neural networks are able to enhance the stability of mobile robot systems, the combination of relevant neural networks and mobile robots has attracted significant interest. Additionally, the self-learning capabilities of neural networks can address sensor interference and external environmental factors, thereby improving localization anti-interference ability. However, current neural network-based algorithms still encounter limitations for localizing in various indoor scenarios. When employing the same neural network algorithm, the mobile robot experiences significant errors in varying environments. Further research should explore the combination of additional sensors and the application of more complex neural network structures to meet the navigation requirements in diverse indoor environments. With the emergence of large-scale language models and advancements in chip technology, neural network-based approaches are expected to rapidly expand in the future.

## Figures and Tables

**Table 1:** The advantages and disadvantages of each sensor and the related representative SLAM work applied for the first time to autonomous mobile robot perception.

Camera Type	Application Time	Characteristic	Advantages and Disadvantages
Monocular Camera	2007 [70]	Contains only one lens, the basic unit of the visual sensor	Pros: easy to integrate, low cost Cons: cannot directly provide in-depth information
Stereo Camera	2008 [71]	Simultaneous generation of left and right images for stereoscopic visual	Pros: produces two images (left and right), and can be used for stereo visual Cons: calibrates camera, computationally complex, limited depth estimation range
RGBD Camera	2011 [72]	RGB images and depth images can be provided directly	Pros: Provides accurate depth information Cons: Depth information is susceptible to environmental influences
Event Camera	2017 [73]	Uses pixel changes as events. Captures luminance changes with microsecond time resolution	Pros: able to cope with high dynamics and scenes with large lighting variations, low power consumption Cons: requires image pre-processing to obtain traditional image information
Light Field Camera	2017 [74]	Adding microlens arrays to camera lenses to add scale information to monocular cameras	Pros: provides scale information for monocular cameras Cons: high computational volume, difficult to achieve real-time computing

**Table 2:** The characteristics of different RF technologies used for indoor localization.

RF	Range (m)	Cost	Advantages	Disadvantages
WiFi	250	high	Implementation simplicity, large coverage, high transmission rate	High power consumption, meterlevel accuracy, vulnerable to NLOS path
Zigbee	100	medium	Extremely low energy consumption, low system cost	Low transmission rate, high latency, vulnerable to NLOS path
Bluetooth	100	low	Implementation simplicity, low energy consumption	Small coverage, low transmission rate, vulnerable to NLOS path
UWB	100	high	High accuracy, extremely high transmission rate, low latency, immune to interference, robustness to NLOS path	High energy consumption and system cost
RFID	5	low	Extremely low energy consumption, implementation simplicity, low system cost, high accuracy with specific approach	Small coverage, vulnerable to NLOS path

**Table 3:** The characteristics of the sensors.

Sensors	Advantages	Disadvantages
IMU	High output frequency, free from environmental interference.	Initial conditions required, error accumulation, accelerometer is susceptible to vibration.
Ultrasonic sensor	Low cost, not influenced by transparent or reflective objects	long-range intensity attenuation, vulnerable to ambient noise.
Infrared sensor	Low cost.	Susceptible to environmental interference (light source, reflection).
LiDAR	High distance measurement accuracy.	Restricted in environments surrounded by transparent or reflective objects.
Vision-based sensor	Rich collection of information	Large computational volume, vulnerable to light.
Radio Frequency Sensor	Environment and obstacles have less effect on positioning.	Not suitable for unknown or unpredictable environments, unable to measure orientation.
